# Evaluation of a new VMAT QA device, or the “X” and “O” array geometries

**DOI:** 10.1120/jacmp.v12i2.3346

**Published:** 2011-01-31

**Authors:** Vladimir Feygelman, Geoffrey Zhang, Craig Stevens, Benjamin E. Nelms

**Affiliations:** ^1^ Division of Radiation Oncology H. Lee Moffitt Cancer Center Tampa Florida 33612 USA; ^2^ Canis Lupus LLC Sauk County Wisconsin 53561 USA

**Keywords:** IMRT QA, rotational therapy, dose distribution comparison, three‐dimensional dosimetry, diode array

## Abstract

We introduce a logical process of three distinct phases to begin the evaluation of a new 3D dosimetry array. The array under investigation is a hollow cylinder phantom with diode detectors fixed in a helical shell forming an “O” axial detector cross section (ArcCHECK), with comparisons drawn to a previously studied 3D array with diodes fixed in two crossing planes forming an “X” axial cross section (Delta^4^). Phase I testing of the ArcCHECK establishes: robust relative calibration (response equalization) of the individual detectors, minor field size dependency of response not present in a 2D predecessor, and uncorrected angular response dependence in the axial plane. Phase II testing reveals vast differences between the two devices when studying fixed‐width full circle arcs. These differences are primarily due to arc discretization by the TPS that produces low passing rates for the peripheral detectors of the ArcCHECK, but high passing rates for the Delta^4^. Similar, although less pronounced, effects are seen for the test VMAT plans modeled after the AAPM TG119 report. The very different 3D detector locations of the two devices, along with the knock‐on effect of different percent normalization strategies, prove that the analysis results from the devices are distinct and noninterchangeable; they are truly measuring different things. The value of what each device measures, namely their correlation with – or ability to predict – clinically relevant errors in calculation and/or delivery of dose is the subject of future Phase III work.

PACS number: 87.55Qr

## I. INTRODUCTION

### A. QA devices evolve with planning and delivery systems

As radiation therapy becomes ever more customizable to each individual patient, the complexities of the supporting treatment planning system (TPS) and the delivery system increase. This, in turn, requires a constant evolution of quality assurance (QA) methods used to verify the performance of the systems. Of course, different types of systems have different QA needs. For example, the following types of X‐ray radiation therapy delivery systems are different enough from each other that they demand unique QA strategies: static gantry intensity‐modulation radiation therapy (IMRT), helical TomoTherapy, volumetric modulated arc therapy (VMAT), and robotic arm therapy.

In the last decade, many QA strategies, only a small subset of which can be cited,^(^ ^1^ ^–^ ^9^ ^)^ have evolved around static‐gantry IMRT, given the uniqueness not only of each patient treatment plan but also of each treatment beam. A common strategy uses per‐beam analysis (measurement vs. TPS calculation) in a single plane in a flat phantom. This strategy was summarized in detail in the AAPM TG 119 report on the IMRT commissioning.^(10)^ However this approach is less than ideal for a rotating beam. As rotational therapy grows in popularity, new QA strategies are emerging, one of which is the use of 3D dosimetry phantoms to allow the entire rotational plan to be delivered to the phantom and the measured dose values compared to TPS calculations on the virtual model of the phantom.

### B. Validation of new QA devices: a process of three distinct phases

In attempt to meet emerging needs, new commercial QA devices naturally arise. Modern devices are typically sophisticated systems comprised of hardware, firmware and software components. It should be noted that in the United States, dosimetry QA devices are most often classified as “Class I” medical devices, and are thus exempt from some of the Good Manufacturing Practice (GMP) and Quality System Regulation (QSR) requirements^(11)^ and are seldom audited for adherence to Design Control Guidance.^(12)^ Additionally, governing professional associations do not prescribe acceptance testing for certain QA equipment. There is no guidance document on the acceptance testing of 3D dosimeters as there is, for example, for accelerator commissioning equipment.^(13)^ As a result, the critical step of design “validation” (assuring the device design satisfies user needs and intended uses^(12)^) has historically been left to the discretion of the end user. Even if the manufacturer completes and documents its own proof of design validation, a medical physicist should properly commission each device in order to learn its limitations, prove its usefulness, and generate procedures for clinical use.

**Figure 1:**
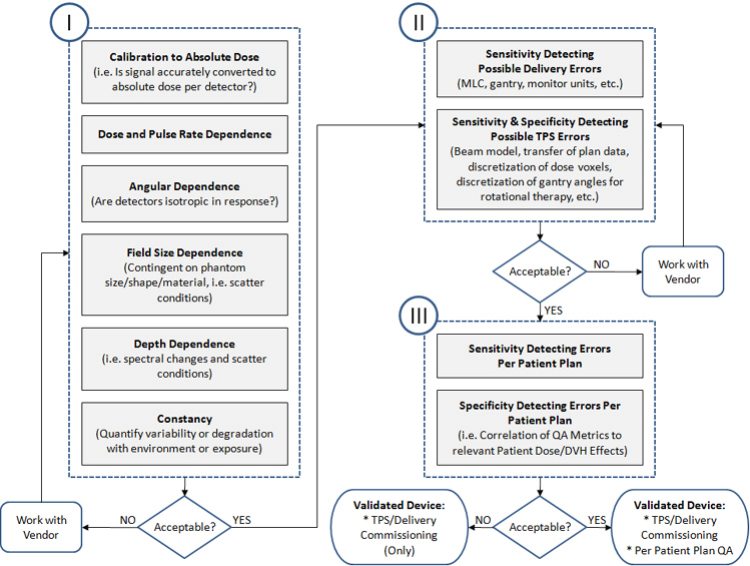
General flow chart for validating a new 3D dosimetry phantom with intended use for: 1) commissioning TPS and delivery systems, and/or 2) per‐patient plan dose QA. There are three serial phases of validation testing.

The intended use of 3D dosimetry phantoms can be generalized into two tasks: 1) commissioning of TPS and delivery systems, and 2) per‐patient dose QA. In order to commission a 3D dosimetry system, a physicist might employ a three‐phased validation testing strategy, summarized as a flow chart in Fig. 1. The purpose of the Phase I validation is to verify that the detectors in the 3D phantom report accurate absolute dose values when irradiated in a range of possible conditions. In this paper, the term ‘absolute dose’ is used to mean that the detector has been calibrated against a standard instrument, such as an ion chamber, to report the readings in Gy. These readings can be directly compared to the absolute dose distribution generated by the TPS, without any renormalization. Phase II validation follows acceptance of the Phase I results, and is geared towards quantifying the performance of the QA device's ability to detect errors in the TPS and/or delivery system (e.g., validating the dosimeter's abilities as a commissioning device). Phase II can have great value in instructing a medical physicist about the limitations of the QA device. Finally, Phase III validation will determine if the QA device is suitable for per‐patient/plan dose QA by quantifying the sensitivity and specificity in detecting clinically relevant errors.

There have been previous studies on 3D dosimetry phantoms, including a high‐density volumetric detector system made of solid gel,^(^ ^14^ ^–^ ^18^ ^)^ radiographic film curved in a spiral pattern,^(19)^ and two orthogonal planes of point detectors with an “X” axial cross section.^(^ ^20^ ^–^ ^23^ ^)^ The purpose of this work was to perform Phase I and Phase II validation testing on a new commercial 3D dosimetry system with an “O” detector arrangement cross section called “ArcCHECK” (Sun Nuclear Corporation, Melbourne, FL). In the process, we illustrate examples of Phase I and Phase II test strategies. We would like to emphasize that, given the high degree of customization of modern radiation treatment plans, the value of next phase of testing – Phase III – is immense, and these studies have also been done using these two devices. However, to maintain focus, this paper addresses only the Phase I and Phase II aspects, which are, after all, required precursors to Phase III.

## II. MATERIALS AND METHODS

All measurements were done with a 6MV X‐ray beam from a Trilogy linear accelerator equipped with a 120‐leaf Millennium MLC (Varian Medical Systems, Palo Alto, CA). The Record and Verify system was Mosaiq v. 1.60 (Impac Medical Systems (an Elekta company), Sunnyvale, CA) and the TPS was Pinnacle v. 9.0 (Philips Radiation Oncology Systems, Fitchburg, WI).

**Figure 2:**
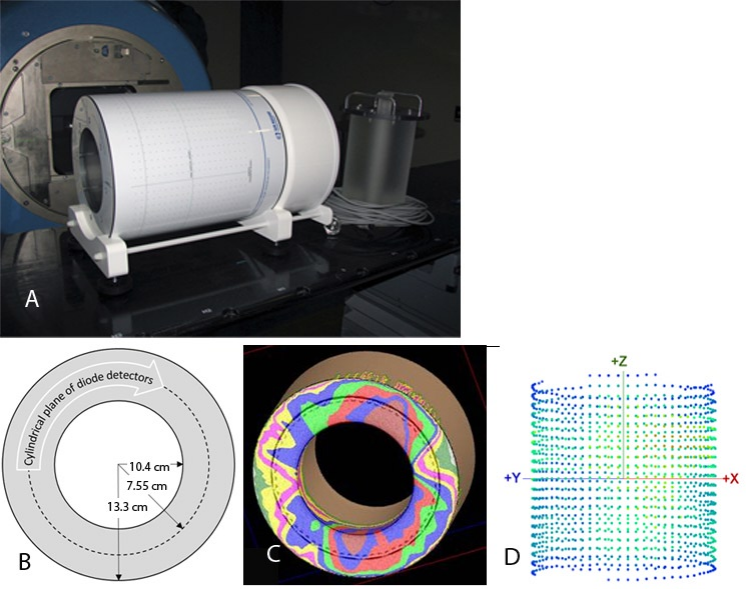
The 3D dosimetry device: (a) overview with an optional PMMA plug on the right; (b) axial cross section of phantom and detector geometry; (c) rotational plan calculated on the virtual model of the device; and (d) 3D view of the diode detector positions, as seen from a 45° gantry angle.

The prototype 3D dosimetry system was described elsewhere,^(^ ^24^ ^,^ ^25^ ^)^ while the commercially available unit that is the subject of this paper is illustrated in Fig. 2. It features a 2D array of 1386‐point detectors that is curved to form a cylindrical surface inside a doughnut‐shaped phantom. The phantom has an outer diameter of 26.6 cm and an inner hole diameter of 15.1 cm, with the curved plane of diodes at a distance of 10.4 cm from the center. The diodes form a helical pattern. They are positioned 1 cm apart along both the cylindrical length and circumference. This detector pattern is presumably designed to accomplish two goals: 1) to reduce the rotational response dependence by making the detector array nearly cylindrically symmetrical, and 2) to increase the apparent detector density in the beam's eye view (BEV) by shifting the exit diodes with respect to the entrance ones. The overall device length is 44.3 cm, of which 11.9 cm is taken up by the electronics section and the remaining 32.4 cm is the length of the PMMA phantom. The active area (detector array) length is 21 cm. The PMMA buildup and backscatter is approximately 2.9 cm each (water equivalent depth $3.3\;{g/\text{cm}}^{2}$). The diode detectors used in this 3D device are the same as described before for a planar array from the same vendor (MapCHECK).^(^ ^6^ ^,^ ^26^ ^)^

The software is capable of comparing the measured and reference dose in either relative or absolute dose and can, among other things, report the gamma analysis^(27)^ results with global or local dose error thresholds.^(^ ^6^ ^,^ ^10^ ^)^

### A. Phase I tests

For dosimetry devices, it is necessary to first investigate how accurately each detector reports absolute dose, and to record any limitations or conditions of failure. In the case of the 3D dosimetry device evaluated here, the dose‐per‐pulse and dose‐rate dependencies were reported in both planar^(^ ^6^ ^,^ ^26^ ^)^ and cylindrical^(^ ^24^ ^,^ ^25^ ^)^ array configurations and are not repeated in this study. We report here the following Phase I tests, in increasing order of importance: detector flip, rotisserie, field size dependence and angular dependence.

#### A.1 “Detector flip test” (symmetry of response after reversal of phantom's long axis)

**Figure 3:**
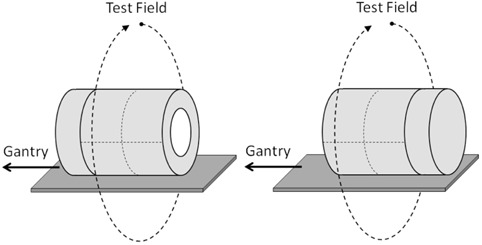
Schematic of the Phase I detector flip test.

A classic relative calibration test is the “detector flip” test.^(26)^ The dosimeter is irradiated typically by a wide field and then rotated by 180° and re‐irradiated by the identical field (Fig. 3). The large field size ensures that all the detectors are in the high dose/low dose gradient area. Ideally, a dosimetry device should produce the same results for both exposure positions. The ArcCHECK is not, strictly speaking, symmetrical with respect to the 180° rotation around the vertical axis due to the helical arrangement of the diodes. However, the dose distributions produced by full arcs are sufficiently uniform to warrant the rotation test, even in the absence of perfect dosimeter symmetry. The maximum spatial displacement of the diodes under a 180° rotation is about 1 mm, verified by rotating the planar overlay of the diode's positions and inspecting. Two arcs were used in the detector flip tests: $10\times 25\;{\text{cm}}^{2}$ and $25\times 25\;{\text{cm}}^{2}$, each spanning 358° and delivering a total of 800 monitor units (MU). Field size naming follows the IEC convention: X direction (parallel to axial plane) × Y direction (parallel to the long axis of the cylindrical phantom). The measured absolute dose data were extracted into a spreadsheet and the relative dose error statistics per detector position were calculated.

#### A.2 “Rotisserie test” (symmetry of response with phantom rotated axially)

**Figure 4:**
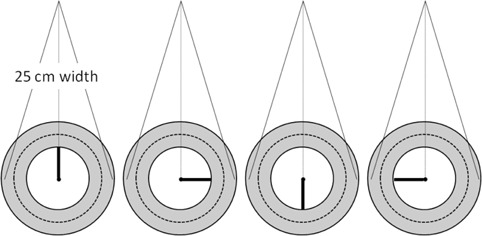
Schematic of the Phase I rotisserie test.

Similar to the detector flip test, the “rotisserie” test is another test of the robustness of the dosimetry device's relative calibration – this time with respect to the incidence angle of a single beam in the axial plane. Similar tests were reported for the prototype^(^ ^24^ ^,^ ^25^ ^)^ but not for the production unit with the full complement of the diodes. Ideally, a dosimetry device should produce the same results for any beam angle exposure. It would be particularly expected for a device such as the ArcCHECK where the detector arrangement is close to being cylindrically symmetrical. For the rotisserie test, an open $25\times 25\;{\text{cm}}^{2}$ static field was delivered with the gantry angle fixed at 0°. The phantom was irradiated four times, rotated in 90° increments between the irradiations (Fig. 4). The absolute dose for each detector was extracted for every measurement and compared.

#### A.3 Field size dependence

Quantifying the field size dependency of the detector response is especially important for diode‐based dosimeters which are known to deviate from the ion chamber due to the over‐response to low‐energy scattered photons.^(^ ^28^ ^,^ ^29^ ^)^

The ArcCHECK field size dependence was quantified in this work for both the hollow and plugged (full backscatter) phantom configurations. With the ArcCHECK centered on the lasers, the array was rotated slightly around the y‐axis to bring one of the central diodes exactly to the center of the radiation field. The readings for two diodes (± 5.5° rotation) were averaged. The measurements were for a range of field sizes from $5\times 5\;{\text{cm}}^{2}$ to $25\times 25\;{\text{cm}}^{2}$ and were normalized to the 10 cm×10 cm field. No change in the diode response relative to the ion chamber is expected for the fields between 5×5 and $2\times 2\;{\text{cm}}^{2}$.^(30)^ Because the ArcCHECK does not have holes drilled for ion chambers in the plane of detectors, the corresponding ion chamber readings were instead obtained with a Farmer chamber in a standard $30\times 30\;{\text{cm}}^{2}$ rectangular Plastic Water phantom (CIRS Inc., Norfolk VA), with 3.3 cm buildup and backscatter, approximating the ArcCHECK geometry. The chamber was placed at the 89.6 cm source‐to‐detector distance (SDD), corresponding to the top diode SDD when the ArcCHECK is centered on the room lasers. In the second experiment, the same SDD and buildup were used but the backscatter thickness was increased to 15 cm (full backscatter conditions emulating the plugged ArcCHECK). For completeness, the results were compared with the MapCHECK device irradiated under the same conditions. Two scatter configurations were used for the MapCHECK. One approximated the ArcCHECK geometry without the plug. 1.3 cm of Plastic Water was added on top and 0.6 cm on the bottom, resulting in 3.3 cm water equivalent buildup and backscatter. This arrangement was supported by 15 cm of Styrofoam. The second configuration approximated the ArcCHECK with the plug, achieved by increasing the backscatter to 15 cm while maintaining the same buildup and SDD.

#### A.4 Angular dependence

##### A.4.1 Small angles along longitudinal axis

The longitudinal angular dependence was evaluated in this work to ensure that the beam divergence would not cause differing response. To obtain the baseline, a Farmer chamber was positioned at the isocenter with its axis coinciding with the gantry rotational axis, in the phantom simulating the ArcCHECK scatter conditions. The gantry was rotated from 0° to the maximum angle of 6.4°, corresponding to the greatest possible incidence angle for the diode at the edge of the field when the ArcCHECK is centered on the room lasers. The readings with the symmetrical gantry angles were averaged and normalized by the reading at 0°, to obtain the relative dose at the measurement point for each gantry angle. Next, the ArcCHECK was positioned on the treatment table with the top central diodes at the isocenter height. The table was rotated 90°. The two central diodes thus coincided with the gantry rotational axis. The average reading of those two diodes was used. The readings for the gantry angles symmetrical with respect to the vertical were averaged, normalized to the reading at 0° and compared with the ion chamber data.

##### A.4.2 Angular dependence in the axial plane

With the ArcCHECK design, a narrow beam segment can be considered approximately normal to the detector surface if: 1) the device is aligned with the isocenter, and 2) the segment is positioned approximately on the central axis (CAX). However, as MLC segments deviate away from the CAX and/or as field widths increase, beamlets start to intersect detectors at increasingly tangential angles. For large volume targets, the angular dependence of the ArcCHECK could become an important factor, and it must be accounted for.

**Figure 5:**
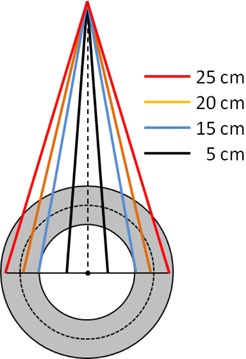
Schematic of the angular dependency tests using beam divergence to generate varying incidence angles. This also represents the setup for the field size dependence test.

A simple experiment to test the angular dependence of the ArcCHECK detectors is to compare the measured and calculated dose profiles for a series of fields with increasing width, looking closely as the field width nears the diameter cross section of the curved detector plane (Fig. 5). A series of open vertical fields ranging in size from $10\times 10\;{\text{cm}}^{2}$ to $25\times 25\;{\text{cm}}^{2}$ was projected onto the ArcCHECK centered on the lasers. The reference 3D dose grids were calculated using 2 mm dose voxels, which is adequate to capture the shape of the beam penumbra. The dose profiles along the curved plane of detectors were normalized to the respective CAX dose. Given the proven accuracy of the TPS calculation in homogeneous phantoms, any substantial deviation in measured vs. calculated profiles would highlight angular‐dependent errors in the measured relative dose. To further justify using the calculated dose as the benchmark for this test, we quantified the agreement between the measured and calculated relative doses in geometry similar to the ArcCHECK using a Solid Water 30 cm diameter cylindrical “Cheese” phantom (TomoTherapy Inc., Madison, WI) and an ion chamber (Exradin A1SL, Standard Imaging Inc., Middleton WI). The chamber was inserted at the 4 cm distance from the surface (11 cm from the center). For the purposes of this test the chamber location is representative of the ArcCHECK diodes positioned 10.4 cm from the center at the 3.3 cm water‐equivalent depth. The gantry was kept vertical and the phantom was rotated in 10° increments to generate measured dose profiles that were normalized to the 0° reading. All relative dose profiles – ArcCHECK measured compared to TPS calculated on the ArcCHECK phantom, and ion chamber measured compared to TPS calculated on the Cheese phantom – were plotted as a function of an angle (ArcCHECK “X” coordinate around the curved surface, which is an angle expressed as a length along a 10.4 cm radius circle).

#### B. Phase II tests

Because the ArcCHECK has such a unique detector arrangement, it is imperative to compare it to a previously validated 3D dosimetry system. One such system with the same intended use is Delta^4^ (ScandiDos AB, Uppsala, Sweden). It was previously thoroughly evaluated by different groups in conjunction with step‐and‐shoot IMRT,^(^ ^20^ ^,^ ^22^ ^)^ VMAT,^(^ ^20^ ^,^ ^31^ ^,^ ^32^ ^)^ and helical TomoTherapy.^(21)^ Among other tests, a diode and film were inserted into the phantom next to the detector boards to independently verify absolute and relative dose agreement.^(^ ^21^ ^,^ ^23^ ^)^ The Delta^4^ contains 1069 diodes positioned on the two orthogonal boards. The detectors are spaced at 0.5 cm in the central $6\times 6\;{\text{cm}}^{2}$ region of each board and at 1 cm elsewhere in the $20\times 20\;{\text{cm}}^{2}$ active area. The boards are inserted in the 22 cm diameter PMMA phantom. Since the Delta4 is well characterized and has a detector arrangement (cross‐sectional “X”) so different from the ArcCHECK (cross sectional “O”), it is particularly well suited to elucidate any differences due primarily to the detector geometry.

##### B.1 Detector density effect

###### B.1.1 Abutting MLC segments

One of the most sensitive tests of leaf‐end penumbra modeling examines the profiles through the junction of the two abutting rectangular MLC‐defined apertures.^(33)^ Because this test relies on detector density to detect small spatial errors, it is a good example of a Phase II test on detector density. In this work, the effects of changing the leaf offset from −0.9 to 0.9 mm on the measured dose profile parallel to the gantry rotational axis were studied. Two parallel‐opposed beams, each consisting of two equally weighted rectangular segments abutting at the isocenter, were delivered to both the Delta^4^ and ArcCHECK phantoms. The collimator was rotated 90°. The Delta^4^ phantom was shifted longitudinally by 3 mm to avoid the central detector being at the nominal field junction location. The relative dose profile for the central diode line (Y direction) on the main detector board was acquired. In the case of the ArcCHECK, the phantom was centered on the room lasers, and two profiles in the Y direction were acquired: one through the CAX point of the beam entrance at the 90° gantry angle and another at 270°. The profiles were compared to the XV2 film (Eastman Kodak, Rochester, NY) measurements at 10 cm depth, with the MLC offset ranging from 0 to–0.9 mm. This range of the MLC offsets, commonly referred to as radiation field offset (RFO), covers the span of values reported in the literature to compensate for the difference between the optical and radiation field edges for the rounded‐end MLC leaves.^(34)^

###### B.1.2 Simulation of “split‐field” IMRT

The “split field” IMRT situation is encountered with the current Varian linac when the target volume is large enough to require the Millennium MLC leaf to extend more than 15 cm from the corresponding fixed jaw. Since this violates physical machine constraints, a TPS typically splits the offending field into two or more abutting/overlapping daughter fields, allowing the machine jaws to move between the fields. This is one of the more challenging dosimetric situations, as the inevitable dose errors in the leaf‐end penumbra are magnified because of the repeated junction of the segments along the same transverse lines in the overlap region of the daughter fields. While this problem is not explicitly addressed in the guidance documents on IMRT commissioning,^(^ ^5^ ^,^ ^10^ ^)^ in fact it is one of the better “stress tests” of the quality of the TPS commissioning and also of a detector array's density.

**Figure 6:**
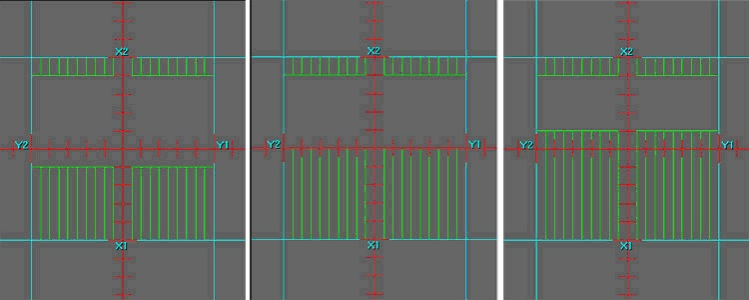
MLC segments comprising the upper half of the split beam used in the split‐filed IMRT test. The overlap region is spread ± 1cm around the central axis. The bottom half segments are a mirror image with respect to the central axis.

We generated a simple plan simulating a typical Varian split field scenario with the segment arrangement depicted in Fig. 6. The overlapping “half‐beams” were symmetrical about the CAX, and each was comprised of three segments shrinking progressively by 1 cm on the overlap side. All six segments were delivered twice as parallel‐opposed beams with the gantry at 270° and 90°.

A number of plans were calculated and measured with the introduction of the systematic MLC shifts of ± 0.3, 0.7 and 0.9 mm for all open leaves. Again, this is the magnitude of the systematic MLC shifts one could find in the real world with the incorrect RFO used during commissioning of a TPS. The measurements with both the Delta^4^ and ArcCHECK were compared to the plans calculated with the same MLC offset, and the gamma analysis values were recorded. Given the submillimeter changes in the MLC positions, all calculations were done on a very fine (1 mm) grid. Then the dose differences between the measurements with and without MLC offset were analyzed. The dose difference is more appropriate than the gamma analysis when both datasets consist of the relatively coarsely spaced measurement points; hence, the distance‐to‐agreement analysis was disabled for these tests.

##### B.2 Dosimetry of full arcs with fixed apertures

Since the purpose of this paper is the evaluation of a new 3D dosimetry phantom with the emphasis on VMAT QA, a baseline of performance should be established by delivering simple full arcs of varying widths and covering, at some dose level, the entirety of detectors.

**Figure 7:**
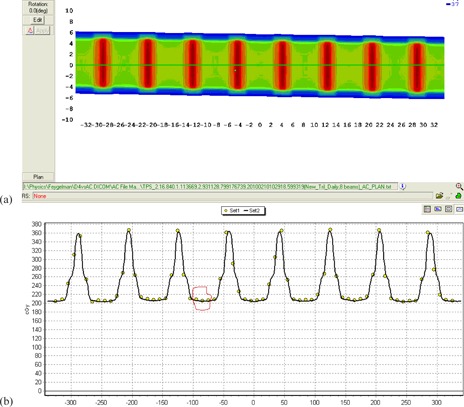
A dose distribution from the eight $10\times 10\;{\text{cm}}^{2}$ beams (a) and a profile along the ArcCHECK X direction (b) illustrating the detector selection for the output correction procedure. The three detector rows in each valley were used; one such set is circled for clarity. The circles on (b) represent diode readings, and the solid line is the dose profile predicted by the TPS.

To eliminate the effects of the accelerator rotational output variations and couch modeling errors, each device was cross‐calibrated prior to the measurement session. Such cross‐calibration is similar in concept to the “plan‐specific reference field” calibration.^(35)^ The Delta^4^ was irradiated by a full $10\times 10\;{\text{cm}}^{2}$ arc. The daily correction factor was determined by a built‐in software routine that minimizes the difference between the measured and calculated dose in the central $6\times 6\;{\text{cm}}^{2}$ region. For the reasons discussed below, an arc‐based cross‐calibration is not recommended for the ArcCHECK. Instead, eight static parallel‐opposed $10\times 10\;{\text{cm}}^{2}$ beams spaced in 45° angular intervals were used. The measured and calculated doses were extracted into a spreadsheet for the detector rows central‐most in the valleys of the ArcCHECK X dose profile. The extracted data were confined to the central 6 cm of the measurement area in the longitudinal (Y) direction, similar to the Delta^4^ cross‐calibration (Fig. 7). A multiplicative correction factor was calculated to minimize the difference between the measured and calculated dose, and was subsequently used as a daily output correction factor.

###### B.2.1 Field widths 5 cm and above

This test was designed to compare agreement between measured and calculated dose for wide open arcs, which produce relatively uniform dose distributions in the center of a cylindrical phantom. Simple 5×25,10×25, and $25\times 25\;{\text{cm}}^{2}$ full arcs (181° to 179° clockwise, IEC1217 gantry angle conventions) were used. The dose was calculated by the TPS at 2° angular increments at a 3 mm dose grid resolution (which was previously proven adequate theoretically^(36)^ and empirically^(31)^) for the Delta^4^ and at a 2 mm dose grid resolution for the ArcCHECK. The finer 2 mm resolution was chosen for the ArcCHECK to eliminate any doubt that the higher dose gradients anticipated at the diode locations are faithfully represented,^(36)^ while maintaining reasonable calculation times. The table top was included in the calculations as a region of interest (ROI). For establishing the percent dose error normalization value, a different method was used for each device given the different arrangement of detectors. The Delta^4^ results were normalized at the isocenter, while the ArcCHECK results were normalized to the maximum measured dose in the detector ring. Since the arc doses are expected to be uniform in the center of the measurement area for both devices, this normalization point selection does not favor either one, but it is worth noting this difference for subsequent discussion.

###### B.2.2 Field widths 2–5 cm

The fixed field arc tests with narrower apertures allow a very unique analysis of 3D dosimetry phantom detector arrangements, as they quantify the effect of the TPS discretization of continuous arcs into static beams, a method employed by the current TPS. The effects of discretization are amplified with increasing radial distance from the isocenter,^(37)^ leading to the dose errors in the periphery that can be potentially vastly different than the errors near the phantom center. This effect, stemming from the “small arc approximation”,^(37)^ is vital to study when considering the peripheral detector location in the ArcCHECK. In order to quantify how dose differences due to angle discretization can be expected to vary by subvolume, we performed a computer simulation. We calculated a $2\times 10\;{\text{cm}}^{2}$ arc at 2°, 4°, and 6° control point (CP) angular spacing and filtered the dose comparisons analysis by subvolumes of solid central cylinders (1, 3, 5, and 10 cm diameter) and the ArcCHECK detector plane approximated by a 2 mm thick ring of 10.4 cm average radius. Percent dose differences were calculated in two ways: 1) global normalization (normalized to maximum dose per subvolume), and 2) local normalization (normalization per voxel based on that voxel's dose). Comparison statistics per ROI were calculated and analyzed by a 3D dose QA software program (3DVH, Sun Nuclear).

The effect of the field size variation on calculated dose at the detector locations was then studied. The field width was varied by ±1 mm on each side for the arcs described above, and the resulting changes were recorded for the isocenter dose and the average dose at the ArcCHECK detector locations nearest to Y=0.

Finally, actual measurements with the ArcCHECK were performed for the full arcs of 2×10,3×10, and $5\times 10\;{\text{cm}}^{2}$ field sizes. The results were compared with the Delta^4^ measurements. The percent difference normalization of the Delta^4^ and the ArcCHECK were the same as described earlier for the larger field full arcs. All TPS calculations for these tests were done with 2°, 4°, and 6° CP angular increment and 2 mm dose grid resolution.

##### B.3 VMAT dosimetry

We have previously reported^(31)^ the results of dosimetric evaluation of the Philips SmartArc^(38)^ VMAT plans. It used the test plan suite and methodology of the AAPM TG119 report,^(10)^ with obvious modifications for rotational delivery. Dosimetric agreement was well within the report's recommendations. A subset of the previously studied plans was chosen for this work. One single‐arc plan was randomly selected for the Multi‐Target and Mock Prostate. Similarly, one single‐arc and one double‐arc plan were selected for both Mock H&N and C‐shape structure sets.

**Figure 8:**
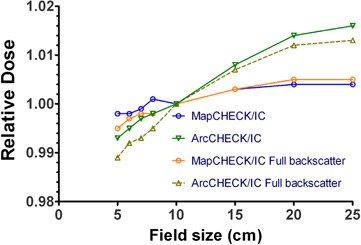
Field‐size dependence variation shown by the ratios of diode to Farmer chamber readings for different field sizes and for different scatter conditions.

Compared to previous work, we improved the measurements by explicitly including the table top in the calculations as an ROI, and by obtaining the daily correction factor for the Delta^4^ from a full arc as opposed to a pair of parallel‐opposed fields. The eight‐field cross‐calibration arrangement described above was used for the ArcCHECK. A field size dependence correction was manually applied to the ArcCHECK results. To that end, an average equivalent square per arc was approximated as a square root of the average MLC opening area reported by the TPS. The correction factors were determined from Fig. 8. They ranged from 0.997 to 0.993. The correction factor was applied uniformly to the entire dose matrix. This is, of course, a rough approximation since these correction factors need to be applied for individual openings at the segment level.

In this paper, we limit the VMAT analysis to the two scenarios. First, we compare the ArcCHECK and Delta^4^ results for the plans calculated on a 2 mm grid but optimized and calculated with different CP increments (2°–6°). Then, we investigate the same plans calculated with a set of parameters that was previously found to be a good practical compromise between calculational speed and accuracy – 3 mm grid and 4° CP increment.

For both the Delta^4^ and ArcCHECK, the gamma analysis values at the 3%/3 mm and 2%/2 mm error levels are reported. Dose error normalization is global for both devices. For the Delta^4^, the prescription dose was used for normalization, in line with the TG119 methodology for the absolute measurements.^(10)^ It is justified because the 22 cm diameter phantom is reasonably similar in size to $20\times 20\times 20\;{\text{cm}}^{3}$ Plastic Water cube used for original planning and ion chamber measurements. For the ArcCHECK, the default normalization – to the maximum measured dose in the curved plane – was used. Because of the detectors’ location away from the isocenter, the prescription dose normalization is not applicable in the ArcCHECK case. It should be noted that the normalization scheme for the ArcCHECK used the highest possible dose in the measurement area, while the maximum dose in the Delta^4^ measurement volume was always higher than the prescription, sometimes by as much as 25%.

### III. RESULTS

#### A. Phase I tests

##### A.1 Detector flip test (symmetry of response after reversal of phantom's long axis)

For the $25\times 25\;{\text{cm}}^{2}$ arc, the mean relative dose deviation between the normal and reverse orientations for all diodes was −0.3±0.2% (range −1% to 0.3%). For the $10\times 25\;{\text{cm}}^{2}$ arc, the mean relative deviation was −0.4±0.4% (range −1.7% to 0.9%).

##### A.2 Rotisserie test (symmetry of response with phantom rotated axially)

The results are presented in Table 1. The deviation from the average is under ± 1%.

**Table 1:** Results of the rotisserie test. Shown are the statistics of the relative dose deviation for all diodes for each of the four rotated phantom positions compared to the average of the four positions.

	*0° ‐ Avg (%)*	*90° ‐ Avg (%)*	*270° ‐ Avg (%)*	*180° ‐ Avg (%)*
Mean	0.3	0.1	0.2	−0.7
Standard Deviation	0.5	0.7	0.6	0.8
99% Confidence Interval	0.3 to 0.4	−0.0 to 0.2	0.1 to 0.3	−0.8 to −0.5

##### A.3 Field size dependence

Unlike with the MapCHECK, the ArcCHECK diode response deviates from the Farmer chamber (Fig. 8). For the hollow phantom, the percentage measured dose difference (ArcCHECK–ion chamber) changes from −0.7% for a $5\times 5\;{\text{cm}}^{2}$ field size to +1.7% for a $25\times 25\;{\text{cm}}^{2}$ field. The corresponding deviations from the ion chamber reading when the PMMA plug is inserted into the phantom are from −1.1% to 1.3%.

##### A.4 Angular dependence

###### A.4.1 Small angles along longitudinal axis

**Figure 9:**
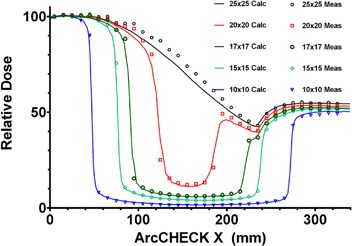
Measured and calculated relative ArcCHECK half‐profiles for field sizes from 10×10 to $25\times 25\;{\text{cm}}^{2}$.

**Figure 10:**
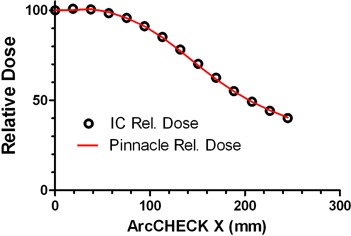
Relative ion chamber and Pinnacle TPS calculated doses in the cylindrical Cheese phantom for a $25\times 25\;{\text{cm}}^{2}$ open field.

Relative diode readings did not vary compared to the ion chamber by more than 0.1%. *A.4.2 Angular dependence in the axial plane* The results of the ArcCHECK response vs. the Pinnacle calculated dose for varying field sizes are shown in Fig. 9 and the comparison of the ion chamber results with Pinnacle in Fig. 10. The largest difference between the measured and calculated dose was observed for the $25\times 25\;{\text{cm}}^{2}$ field – up to 7%. The difference between the ion chamber and TPS relative doses for this field did not exceed 1.3% (mean deviation 0.2%± 0.7%).

#### B. Phase II tests

##### B.1 Detector density effect

###### B.1.1 Abutting MLC segments

**Figure 11:**
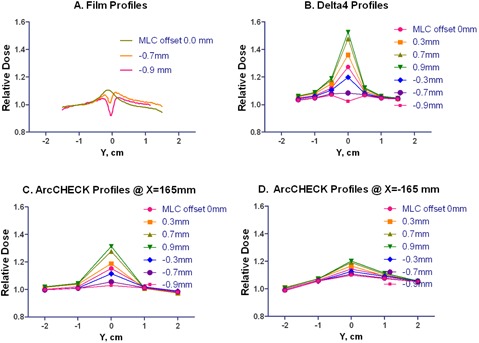
Longitudinal profiles with film, Delta^4^ and ArcCHECK (X=±165 mm). The MLC offset varied from 0.0 to −0.9 mm for the film and from −0.9 to 0.9 mm for the diode arrays. For the ArcCHECK, X = ± 165 mm correspond to the CAX beam entry points for lateral beams.

The dose profiles are presented in Fig. 11. The ArcCHECK results differ between the two X positions, depending on how close the detector locations on the helical grid are to the junction region. The maximum/minimum ratio (0 to −0.9 mm offset) with the high resolution dosimeter (film) is 1.19. For the Delta^4^ it is 1.24. For the ArcCHECK, it is 1.12 for the profile exhibiting the larger spread (ArcCHECK X=165 mm). The ArcCHECK does not register the dip in the center of the profile with the MLC offset of −0.9 mm. The Delta^4^ reproduces it at least qualitatively.

###### B.1.2 Simulation of split‐field IMRT

Table 2 summarizes the gamma analysis results comparing the measured and calculated doses for different introduced MLC offsets.

**Table 2:** Gamma analysis passing rates (%), measured vs. calculated for overlapping split fields with different MLC offsets. The offset is the same for planning and delivery.

	${\text{Delta}}^{4}$		*ArcCHECK*	
*MLC Offset (mm)*	γ(3/3)≤1	γ(2%/2)≤1	γ(3/3)≤1	γ(2/2)≤1
0.0	100	97.7	100	98.0
0.3	98.9	92.2	99.3	97.3
0.7	100	99.0	100	96.8
0.9	99.2	94.5	100	98.0
−0.3	100	100	100	97.5
−0.7	100	97.7	100	99.0
−0.9	100	99.2	99.5	98.2

Table 3 shows the dose difference results for the measurements with different MLC offsets compared to the zero offset measurement.

**Table 3:** Drop in dose error passing rates at two error threshold levels for split‐field plans delivered with different MLC offsets. All compared to the passing rate data with no offset.

	${\text{Delta}}^{4}$		*ArcCHECK*	
*MLC Offset (mm)*	*Percent Drop in* ΔD≤3%	*Percent Drop in* ΔD≤2%	*Percent Drop in* ΔD≤3%	*Percent Drop in* ΔD≤2%
0.3	11.2	29.1	4.5	5.2
0.7	31.1	25.7	9.7	13.7
0.9	38.8	45.0	16.1	19.4
−0.3	7.5	18.7	2.5	4.9
−0.7	31.5	34.0	10.9	11.9
−0.9	33.7	43.9	12.9	19.1

##### B.2 Dosimetry of full arcs with fixed apertures

###### B.2.1 Field widths 5 cm and above

The original gamma analysis results are summarized in Table 4. The field size dependence correction factor for elongated fields was then measured (Table 5) and applied to the ArcCHECK measurements. This resulted in the γ(3%/3 mm) and γ(2%/2 mm) passing rate increase for the $10\times 25\;{\text{cm}}^{2}$ arc to 96% and 83%, respectively.

**Table 4:** Gamma analysis passing rates (%), measured vs. calculated, for the simple arcs.

	${\text{Delta}}^{4}$		*ArcCHECK*	
*Field Size (cm^2^)*	γ(3/3)≤1	γ(2/2)≤1	γ(3/3)≤1	γ(2/2)≤1
5×25	100.0	100.0	97.6	86.1
10×25	100.0	99.7	86.4	62.6
25×25	100.0	100.0	5.8	0.0

**Table 5:** Ratio of relative responses of the ArcCHECK and Farmer chamber in the elongated fields.

*Field Size (cm^2^)*	*ArcCHECK/IC*
5×25	0.999
10×25	1.008
25×25	1.013

###### B.2.2 Field widths 2–5 cm

**Figure 12:**
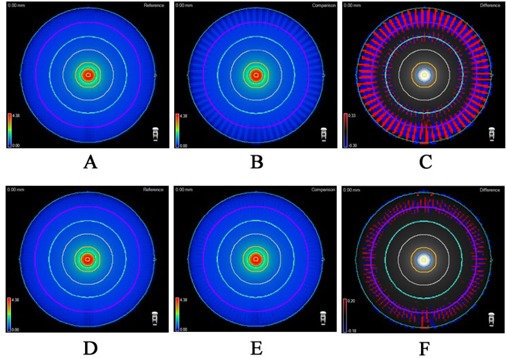
3D comparisons of a simple continuous arc delivery, discretized into static beams for calculation: (a) 2° spacing; (b) 6° spacing; (c) the regions of dose differences > 1% (6° ‐ 2°); (d) 2° spacing; (e) 4° spacing; F) the regions of dose differences > 1% (4° ‐ 2°). The purple ring represents the ArcCHECK detector surface.

**Figure 13:**
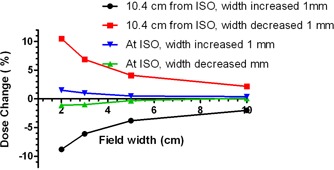
Percent change in calculated dose with the arc width change of 1 mm on each side. Isocenter dose (representative of Delat^4^) compared with the ArcCHECK detector location (10.4 cm from isocenter).

**Figure 14:**
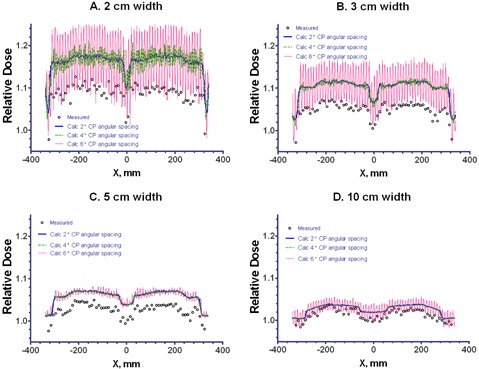
Measured and calculated dose profiles on the curved detector plane for a variety of arc aperture widths. For calculations, dose grid resolution was set at 3 mm and angular discretization varied from 2° to 6°. As evident from the graphs, the calculation dose grid resolution is adequate to demonstrate the discretization effect, while maintaining reasonable calculation times.

Figure 12 graphically illustrates the differences in dose calculated with different CP angular increments on a homogeneous phantom, for a 2 cm wide arc. The 2° CP increment calculation is used as a reference, and the differences for the 4° and 6° CP increments from the 2° are plotted. It is clear that the dose difference increases away from the isocenter. These data are quantified in Tables 6 and 7 in terms of the gamma analysis for different volumes of interest: solid central cylinders progressively increasing in diameter, and a hollow ring simulating the ArcCHECK detector surface. The effect of the small changes in field size on the dose near and away from the isocenter is presented in Fig. 13. The measured and calculated dose profiles in the X direction for different field widths are compared for the ArcCHECK in Fig. 14. The graphs clearly show a sawtooth pattern in the calculated profiles as the CP spacing increases and the filed size decreases. The agreement between the measured and calculated doses decreases as the field width decreases. This is also confirmed by the gamma analysis of the measured vs. calculated dose presented in Table 8, in comparison with the Delta^4^.

**Table 6:** Quantification of the effect of TPS angle discretization (2°, 4° and 6° angular CP spacing) when estimating continuous, full rotation simple 2 cm‐wide arc. All values are in passing rates (%, calculated vs. calculated) with global normalization dose equal to the maximum dose in the corresponding subvolume and a lower threshold dose of 10% of the normalization dose.

	*100% Norm*		$6^{0}-2^{0}$			$4^{0}-2^{0}$	
*ROI Analyzed*	*Dose (Gy)*	γ(3/3)≤1	γ(2/2)≤1	γ(1/1)≤1	γ(3/3)≤1	γ(2/2)≤1	γ(1/1)≤1
1 cm Diameter Cylinder	4.375	100	100	94.4	100	100	100.0
3 cm Diameter Cylinder	4.375	100	99.9	84.6	100	100	96.7
5 cm Diameter Cylinder	4.375	100	100	94.0	100	100	98.9
10 cm Diameter Cylinder	4.375	100	100	98.4	100	100	99.7
ArcCHECK Detector Surface	0.430	56.5	51.3	42.3	70.2	58.5	43.5

**Table 7:** Quantification of the effect of TPS angle discretization (2°, 4° and 6° angular CP spacing) when estimating continuous, full rotation simple 2 cm‐wide arc. All values are in passing rates (%, calculated vs. calculated) with local dose normalization.

		$6^{0}-2^{0}$			$4^{0}-2^{0}$	
*ROI Analyzed*	γ(3/3)≤1	γ(2/2)≤1	γ(1/1)≤1	γ(3/3)≤1	γ(2/2)≤1	γ(1/1)≤1
1 cm Diameter Cylinder	100	97.3	87.2	94.5	92.2	88.0
3 cm Diameter Cylinder	100	98.3	82.2	96.1	93.2	88.9
5 cm Diameter Cylinder	100	98.5	90.0	97.1	94.8	90.3
10 cm Diameter Cylinder	100	99.6	94.3	98.3	96.9	93.4
ArcCHECK Detector Surface	56.6	51.2	42.1	70.0	58.2	40.5

**Table 8:** Passing rates (%), measured vs. calculated, for the smaller fixed arcs.

	${\text{Delta}}^{4}$		*ArcCHECK*	
*Field Size* $\left({\text{cm}}^{2}\right)$	γ(3/3)≤1	γ(2/2)≤1	γ(3/3)≤1	γ(2/2)≤1
2×10	100	99.8	25.1	‐
3×10	100	100	33.3	‐
5×10	100	100	57.8	‐
10×10	100	100	94.8	68.5

##### B.3 VMAT dosimetry

In Table 9, the dose calculation grid was fixed at the lowest practical value – 2mm, while the CP increment was varied within the allowed SmartArc range. The passing rates are numerically slightly different from the previous work^(31)^ due to the improved measurement methodology. As the CP increment changes from 2° to 6°, the Delta^4^ average passing rates decrease by 1.6 and 3.7 percentage points for the γ(3%/3 mm) and γ(2%/2 mm), respectively. The ArcCHECK is subject to a stronger discretization effect, as the corresponding passing rate drop is 4.4 and 10.9 percentage points, respectively.

**Table 9:** Gamma analysis passing rates (%), measured vs. calculated, for VMAT plans optimized with different angular CP spacing and calculated on a 2 mm grid. The fine grid was chosen to isolate the discretization effects.

	${\text{Delta}}^{4}$						*ArcCHECK*					
*Plan*	γ(3,3)≤1			γ(2,2)≤1			γ(3,3)≤1			γ(2,2)≤1		
*CP Increment*	*2°*	*4°*	*6°*	*2°*	*4°*	*6°*	*2°*	*4°*	*6°*	*2°*	*4°*	*6°*
Prostate	100	100	100	98.6	99.6	96.9	99.4	99.4	95.8	95.8	94.3	84.9
Multi‐target	100	100	99.7	99.7	99.2	98.0	98.9	98.6	94.5	93.7	92.4	84.9
H&N, 1 Arc	100	99.5	96.5	98.0	95.3	86.5	99.5	97.4	92.4	94.3	88.5	80.6
H&N, 2 Arcs	100	99.9	98.7	95.3	96.0	91.0	94.2	93.9	91.4	85.8	82.4	78.1
C‐Shape, 1 Arc	99.3	100	94.7	94.4	96.9	87.5	99.6	97.3	94.8	96.6	88.9	78.9
C‐Shape, 2 Arcs	99.9	100	99.9	92.7	99.6	96.8	98.7	96.6	94.9	91.1	86.7	84.7
Average	99.9	99.9	98.3	96.5	97.8	92.8	98.4	97.2	94.0	92.9	88.9	82.0

For the calculation parameters we typically use in the clinic (a 3 mm dose grid and 4° CP increment) both the ArcCHECK and Delta^4^ demonstrate high average passing rates at the γ(3%/3 mm) level (Table 10). However the γ(2%/2 mm) passing rate is lower for the ArcCHECK by 7.6 percentage points. Moreover, while all individual plans in Table 10 exhibit γ(2%/2 mm) passing rates above 92.9% with the Delta^4^ measurement, only two of nine are above 90% with the ArcCHECK.

**Table 10:** Gamma analysis passing rates (%), measured vs. calculated, for VMAT plans optimized with 4° CP spacing and calculated on a 3 mm grid that we typically use in our clinic.

	${\text{Delta}}^{4}$		*ArcCHECK*	
*Plan*	γ(3,3)≤1	γ(2,2)≤1	γ(3,3)≤1	γ(2,2)≤1
Prostate	100	99.6	98.8	94.6
Multi‐target	100	99.2	97.8	90.4
H&N, 1 Arc	98.7	92.9	96.4	87.3
H&N, 2 Arcs	99.9	94.8	95.6	86.5
C‐Shape, 1 Arc	99.7	97.1	97.6	89.3
C‐Shape, 2 Arcs	100	99.7	97.2	89.6
Average	99.7	97.2	97.2	89.6

### IV. DISCUSSION

#### A. Phase I tests

##### A.1 Field size dependence

In the ArcCHECK configuration, the diodes over‐respond as the field size increases. The uncompensated field size dependence can introduce an additional error of about −0.7% for the 5 cm equivalent square segment size and +1.7% for 25 cm. These results demonstrate the expected direction of the difference between the diode and ion chamber measurements. This effect is more pronounced for the ArcCHECK compared to the MapCHECK, which shows negligible filed size dependence, as reported before^(^ ^6^ ^,^ ^26^ ^,^ ^39^ ^)^ and confirmed in this work. In the earlier prototype work,^(25)^ it was tacitly assumed that ArcCHECK would have the same negligible field size dependence as the MapCHECK because it employs the diodes of the same design. However, Fig. 8 clearly illustrates segregation between the ArcCHECK and MapCHECK field size dependence in any scatter configuration. Saini and Zhu^(40)^ suggested that the variation in thickness and design of the buildup around the silicone die could cause differing field size dependence even for the same diode. While determination of the exact reason for the field size dependence difference between the ArcCHECK and MapCHECK is beyond the scope of this work, the empirical data warrant application of the filed size correction to the ArcCHECK measured data.

##### A.2 Angular dependence in the axial plane

The $25\times 25\;{\text{cm}}^{2}$ field is large enough to encompass the full detector profile and thus irradiate detectors at varying tangential angles. Because the ion chamber (Fig. 9) exhibits much smaller dose error than the ArcCHECK (Fig. 10) – 1.3% vs. 7% – the observed differences are attributable to significant angular response dependence of the ArcCHECK detectors. As the field size decreases, especially when it becomes smaller than the detector ring diameter, the angular dependence effect progressively diminishes. The agreement is within 1% for a 15 cm‐wide field, similar to the ion chamber experiment. Since all the arc and VMAT beams used in this work were less than 15 cm in width, the angular sensitivity dependence of the AC detectors did not pose a significant problem. For more general use of the device, the manufacturer is implementing a software angular dependence correction based on the virtual inclinometer concept.^(25)^

### B. Phase II tests

#### B.1 Detector density effect

##### B.1.1 Abutting MLC segments

The proximal diodes in the ArcCHECK have an effective BEV spacing of 1.12 cm as they are typically situated at an 89.6 cm distance from the source. This is too coarse to guarantee that the finer dose profile details are always sampled adequately. The helical shift of the detector pattern does not automatically improve the situation, although the symmetrical parallel‐opposed beam arrangement used in this study assures that on one side of the ArcCHECK the diodes would be closer to the junction line than on the other. Nevertheless, the dip in the middle of the profile with the −0.9 mm MLC offset is not apparent at either left or right side of the ArcCHECK ((Figs. 11c) and (d)). Comparatively, the Delta^4^ follows the film results more closely in the central $6\times 6\;{\text{cm}}^{2}$ region, where the diodes are situated on a 5 mm grid (Fig. 11(b). Although neither device has resolution comparable with film,^(41)^ the 5 mm detector spacing in the center of the Delta^4^ may be the coarsest pitch still adequate to represent realistic IMRT dose gradients.^(9)^ The clinical implications of the detector spacing and location require further investigation (Phase III testing).

##### B.1.2 Simulation of “split‐field” IMRT

The ArcCHECK measurement agrees very well with the calculated reference dose, as does the Delta^4^ (Table 2). However the ArcCHECK is less sensitive to the systematic MLC offset. Comparatively, the Delta^4^ is particularly more sensitive at the 2% dose error threshold level, as its passing rates change by at least 18% with the smallest offset (± 0.3 mm) while the same offset produces changes in passing rate of about only 5% for the ArcCHECK (Table 3). This is strictly a function of detector density.

#### B.2 Full arcs with fixed apertures

##### B.2.1 Field widths 5 cm and above

Delta^4^ shows high agreement with the calculated dose for the simple open arcs. In addition to the results in Table 4, at least 98% of the points pass the γ(2%/1 mm) test for every field size. The ArcCHECK shows a reasonable agreement at the γ(3%/3 mm) level for the $5\times 25\;{\text{cm}}^{2}$ arc, but for the other two experiments the agreement is poor. For the widest arc $\left(25\times 25\;{\text{cm}}^{2}\right)$, the error is caused primarily by the uncompensated angular dependence for the wide beams (Fig. 7). An additional error is introduced by the uncorrected field size dependence (Fig. 8). For the $10\times 25\;{\text{cm}}^{2}$ arc, the γ(3%/3 mm) passing rate improved by about 10 percentage points to 96% upon application of the field size correction, while this correction is negligible for the $5\times 25\;{\text{cm}}^{2}$ (equivalent square close to $10\times 10\;{\text{cm}}^{2}$). Further investigation is needed to determine exactly why ArcCHECK does not show better than 3% dose agreement for the 10 and 5 cm‐wide arcs. One of the possible reasons is detector positioning away from the phantom center, as discussed below.

##### B.2.2 Field widths 2–5 cm

The ArcCHECK detector geometry is presumably based on the assumption that by capturing the entrance and exit dose, one can reliably determine the dose around the isocenter, which is typically of most interest. This assumption should work well for static beams, as a TPS can calculate the depth dose fairly accurately. In fact, a very similar approach was implemented for static gantry in the optional Delta^4^ volumetric calculation.^(23)^ There, the dose at any point along a ray traced from the source through the phantom is determined by renormalizing the depth‐dose distribution known from the TPS, to fit the two measured point dose values at the intersection of the ray with the detector boards. However, a ring detector arrangement has yet to be validated for rotational therapy. At the heart of rotational dose calculations (whether employing simple, conformal, VMAT or tomotherapy arcs), is discretization of the continuous arc into a number of static beams. Webb and McQuaid^(37)^ called this a “small arc approximation”, as applied to VMAT, and pointed out that the robustness of this approximation progressively diminishes as the point of interest moves away radially from the isocenter.

In this paper, there is substantial evidence presented (Tables 6–8 and Figs. 12–14) illustrating that the ring detector arrangement in the ArcCHECK is challenged when evaluating the differences between measured and calculated dose distributions produced by the narrow fixed‐width arcs. This is because the reference (calculated) dose away from isocenter is unstable with respect to the discretization effects (CP angular spacing). The central region's dose is more stable. As shown in Tables 6 and 7, for a 2 cm‐wide arc, there is relatively little difference between the dose distributions calculated with various CP angular increments, as long as the analyzed ROI is a solid cylinder coaxial with the phantom. With global normalization, essentially every voxel agrees to within 2%/2 mm between the 2° and 6° calculations, for any cylindrical ROI from 1 to 10 cm in diameter. Only 51% of the voxels on the simulated ArcCHECK detector surface show the same level of agreement.

Small arc widths present additional challenge. With the nominal beam width of 2 cm, the calculated dose at the ArcCHECK detector location varies by about 10% per 1 mm in the field edge position change (Fig. 13). This translates into 3% dose variation per 0.3 mm of the field edge displacement, which is a value approaching the realistic limits of the MLC accuracy and precision. This instability can lead to substantial apparent dose errors reported by the ArcCHECK while the Delta^4^, which is better geared towards sampling of the central dose, demonstrates robust dose agreement for the fixed arcs regardless of the field width (Table 8). The field size correction was applied to the ArcCHECK measured doses but failed to improve the results in Table 8 appreciably.

It is noted that the low passing rates measured by the ArcCHECK are legitimate given the location of the detectors in the phantom. The discretization of the arc into static beams used by the TPS is a simplification to make the speed of dose calculation practical, and this leads to imperfections in the calculation as one moves outward from the isocenter. ArcCHECK would thus make an excellent commissioning device to study dose effects in the periphery due to TPS discretization approximations. However, for typical patient QA, it is important to know the errors overlapping critical volumes, and the discretization does not appreciably compromise dose calculation centrally.

#### B.3 VMAT plans

Even with the different planar detectors – such as film, the diode array^(6)^ and the ion chamber arrays,^(^ ^8^ ^,^ ^42^ ^)^ to name the most popular ones – analysis results (measured vs. calculated) cannot be readily compared between devices due to differences in detector resolution (detector active area) and detector density (number of detectors per area). However, even though passing rates may differ to some degree between different dosimeters, a user can intuitively compare regions and patterns of error on the analyzed plane, particularly when the detector design differences are accounted for.^(39)^ The dose error definition (local vs. global normalization) can also have a profound effect on the numerical results.^(10)^ Comparisons between the 3D dosimeters are even more complicated when they have vastly different detector geometries, as is the case between the ArcCHECK and the Delta^4^. They not only register the dose in different locations, but the dose error normalization cannot be the same.

Although the VMAT results are not as clear‐cut as the simple arc data, certain conclusions can be made. The ArcCHECK is again more sensitive to the angular discretization effect. However, this effect is mitigated by the varying VMAT beam apertures, compared to the fixed‐width arc. ArcCHECK produced somewhat lower gamma analysis passing rates compared to the Delta^4^, particularly at the 2%/2 mm error threshold level. Based on the fixed‐width arc results discussed earlier, the trend in Table 9 and the small arc approximation theory,^(37)^ it appears likely that at least some of this difference is attributable to the angular discretization of the arcs. However there are other effects besides pure detector geometry that could contribute to this difference. Difference in normalization of the dose error was mentioned before. Also, the 10% dose cut‐off threshold below which the voxel is excluded from analysis may not mean the same on the ArcCHECK diode surface as it does on the Delta^4^ diode plane. Finally, the effect of the central hole inhomogeneity on the VMAT exit dose was not addressed in this work. Due to the symmetry of the opposed‐beam arrangement and, by extension, of the full fixed‐width arc, the inhomogeneity effect is essentially accounted for by cross‐calibration described in the Methods section above. As the symmetry is lost in a modulated plan, an additional error of perhaps 1%–2%^(43)^ could be expected for the exit detectors. This error would likely be TPS‐specific, as different algorithms may handle the low density heterogeneity somewhat differently.

### C. Future Phase III work

Even if the individual diodes in both ArcCHECK and Delta^4^ were ideal detectors, the dose error analysis between the two devices would not be interchangeable due to the different detector placement. This underscores the need for Phase III testing, which will help determine the correlation of device comparison metrics to clinically‐relevant patient dose metrics. Vendors and users may argue about the optimal placement of detectors in a 3D phantom but, in the end, it is Phase III testing that quantifies the “specificity” of the device's response, which can in this context be defined as the ability to predict errors of importance on a per‐patient plan basis. This must be quantified by studying correlation between the device's comparison metric(s) and critical patient dose metrics such as, for example, changes in PTV coverage, parallel organ mean dose or serial organ maximum dose. We have begun this work in earnest using blind studies of induced errors in 3D patient dose. One subject of great interest is to see whether a 3D dosimeter could be proven effective in per‐patient dose QA though be limited as a commissioning device; or, put another way, if a device could excel in Phase III testing though not in Phase I or II. The reverse of that – a device good for commissioning but not sensitive/specific for per‐patient QA – is also possible. Ultimately, one would like a device which provides an accurate and comprehensive toolset for commissioning of the TPS and delivery systems and also produces clinically‐relevant performance metrics useful for per‐patient dose QA.

## V. CONCLUSIONS

We break down QA dosimeter validation into a logical process of three distinct phases (I‐III).

In Phase I testing, the ArcCHECK exhibited robust response uniformity between the diodes. Measurement accuracy for the fields exceeding approximately 15 cm in width is compromised by the diodes’ angular response dependence. This is being addressed by the manufacturer. ArcCHECK exhibits stronger field size response dependence compared to its predecessor, MapCHECK, which should be corrected in the software.

In Phase II testing, the ArcCHECK was found to have limitations for dosimetry of the fixed‐width arcs inherent in the curved detector plane placing all the diodes in the periphery. It has demonstrated good gamma analysis passing rates for the VMAT plans at the 3%/3 mm dose error threshold level. However, the passing rates at the 2%/ mm level were considerably lower compared to the previously evaluated biplanar 3D diode array. At least part of this difference between the two devices is due to composite dosimetry using vastly different detector geometry. Direct comparison of the measured vs. calculated dose passing rates is therefore not feasible. Phase III validation – a study of the device's ability to catch realistic and clinically relevant dose errors – is the subject of future work. Also, ArcCHECK is capable of time‐resolved measurements which were not studied.

## ACKNOWLEDGMENTS

No financial support was received for this study. The authors are grateful to Sun Nuclear Corp. for loaning the ArcCHECK for data collection.
